# Determination of geochemical parameters that control the spatial distribution of potentially toxic elements released from excavated shale at a temporary storage pit

**DOI:** 10.1007/s10653-025-02577-8

**Published:** 2025-06-10

**Authors:** Shoji Suzuki, Toshihiko Miura, Kenichi Ito, Masahiko Katoh

**Affiliations:** 1 Department of Agricultural Chemistry, Graduate School of Agriculture, Meiji University, 1-1-1, Higashimita, Tama, Kawasaki, Kanagawa 214-8571 Japan; 2 Technical Research Institute, Obayashi Corporation, 4-640 Shimokiyoto, Kiyose-Shi, Tokyo, 204-8558 Japan; 3 Center for Collaborative Research and Community Cooperation, The University of Miyazaki, 1-1, Gakuen Kibanadai-Nishi, Miyazaki, Miyazaki 889-2192 Japan; 4 Department of Agricultural Chemistry, School of Agriculture, Meiji University, 1-1-1, Higashimita, Tama, Kawasaki, Kanagawa 214-8571 Japan; 5 Technical Research Institute, Okumura Corporation, 387 Ohsuna, Tsukuba, Ibaraki 300-2612 Japan

**Keywords:** Amorphous iron, Colloid, Estimation error, Excavated rock, Spatial distribution

## Abstract

Large quantities of rocks naturally containing potentially toxic elements (PTEs) are generally excavated from urbanized and mountainous areas for construction purposes and are subjected to treatments (e.g., chemical immobilization) so that they can be re-used. To design such treatments, it is imperative to determine the total PTE amounts released from the excavated rocks and consider their spatial distribution. Within this context, the present study aimed to clarify (i) the spatial distribution of PTE contents released from 100 m^3^ of excavated shale deposited in a temporary storage pit, and (ii) the geochemical parameters that control such distribution. A total of 50 shale samples were collected from a temporary storage pit of rocks. These samples were subjected to the analysis for the determination of the released and total PTE amounts, and other chemical properties. No linear relationship was detected between the released and total PTE amounts. An error of ± 30% was estimated based on a sample of 10 rocks. The geochemical parameters controlling the PTE contents released from the excavated shale differed depending on PTE types. The release of arsenic and selenium was mainly controlled by the amount of amorphous iron, while that of lead and chromium was controlled by the amount of inorganic colloidal particles with size under 0.45-μm. This study suggested that the preparation of 10 mixed rock samples collected from temporary storage pits leads to an estimation of released PTE amounts with a 30% error. In addition, the actual PTE amount released from excavated rocks containing high and low amounts of amorphous iron and inorganic colloidal particles, respectively, would differ little from the estimated amounts.

**Supplementary Information:**

The online version contains supplementary material available at 10.1007/s10653-025-02577-8.

## Introduction

Potentially toxic elements (PTEs), such as arsenic, selenium, lead, and chromium, are widely distributed in the geoenvironment and are toxic to both humans and animals if ingested in excess (Bhattacharya et al., 2015; Fallahzadeh et al., 2018; Hammond, 1977; Moreira et al., 2018; Rahman et al., 2015). Many cases of groundwater containing PTEs released from solid materials (such as sediments) have been reported, causing widespread concern (Berg et al., 2001; Naujokas et al., 2013; Nickson et al., 2000; Pi et al., 2015). Thus, understanding the mechanisms underlying the release of PTEs naturally contained in solid materials is important.

Large volumes of rocks and/or sediments are excavated from urbanized and mountainous areas to clear underground spaces needed for the construction of modern and high-speed railways and roads worldwide (Tabelin et al., 2018). These excavated materials generally contain PTEs at the same concentrations as the background levels or slightly higher. PTEs stably exist in the rocks/sediments before excavation. However, the excavation process increases their solubility on the rock/sediment surface due to physicochemical changes (Tangviroon et al., 2017). For example, the alteration of arsenic to soluble phases induces its release from the excavated materials at rates exceeding the environmental standards for water, i.e., 0.01 mg L^−1^ (Kamata & Katoh, 2019). Thus, such naturally occurring PTE-containing excavated rocks need to be treated appropriately before being re-used. One of the techniques adopted to minimize the risk of PTE leakage into the outer environment is chemical immobilization, which can make the PTE phases more insoluble through reaction with the immobilization material (Katoh et al., 2015a, 2015b; Ogawa et al., 2016; Shimizu et al., 2018).

Determining the total amount of PTEs released from excavated rocks is the first requirement for preparing adequate chemical immobilization treatments. On tunnel construction sites, the amount of released PTEs is generally evaluated for every hundred to thousands m^3^ of excavated rocks, depending on the scale of construction. The solid materials such as soil, sediment, and rock naturally contain the PTEs (Dietrich et al., 2016), and the PTEs have a heterogeneous distribution (Fallazhzadh et al., 2018; Liu et al., 2020; Mishra et al., 2020; Hu et al., 2021). Prince and Pichler (2006) reported that arsenic is present in the Suwannee Limestone in low concentration, but is concentrated in minor mineral phases such as framboidal pyrite. The PTEs naturally contained in solid materials have a heterogeneous distribution (Fallazhzadh et al., 2018; Liu et al., 2020; Mishra et al., 2020; Hu et al., 2021). Vongphutohne et al. (2017) showed that the amount of total arsenic in unconsolidated sediments collected via vertical borings ranged from 3 to 9 mg kg^−1^. Previous knowledge was accumulated from samples collected from a wide area of several km^2^. However, it is not clear whether the spatial distribution of total PTE amounts in several hundred to thousand m^3^ of excavated rock is greater than that over a wide area.

In addition to the total PTE amounts, the amounts released from the excavated rock also have the spatial distribution. Nakajima et al. (2024) collected the excavated rocks including mudstone, sandstone, tuff/tuff breccia, andesite, basalt in Japan, and evaluated the amounts of arsenic released. They reported that arsenic is likely released above the Japanese environmental quality standards (0.1 mg kg^−1^) regardless of rock type. This implies that PTEs may be released regardless of rock type and that the release has the spatial distribution. However, the spatial distribution of the amounts released from the excavated rock is difficult to determine. PTE release is mainly controlled by sorption/desorption and precipitation/dissolution processes, which are both greatly influenced by pH, the oxidation–reduction potential (Eh), dissolved oxygen, and coexisting ions (O’Day et al., 2004; Savage et al., 2000; Tabelin & Igarashi, 2009; Tabelin et al., 2012, 2017; Yang et al., 2015). For example, pH variations alter the charge balances on the surface of iron oxyhydroxide that can sorb PTEs, resulting in the enhancement/suppression of PTE release (Tabelin et al., 2012). The presence of calcium and magnesium ions in the solution increases the sorption of arsenic on the surfaces of excavated rocks (Suzuki & Katoh, 2020). Furthermore, under reducing conditions, arsenic is released from the surface of iron oxyhydroxide during its dissolution (Kamata et al., 2022). The previous knowledge allows us understand that the spatial distribution of PTEs released during excavation depends not only on their total amounts, but also on their phases and the physicochemical properties of excavated rocks. Thus, the spatial distribution of released PTEs may be wider than that of their total amounts in the excavated rocks. In order to accurately determine the total amount of PTEs released from the rock that excavated with the huge amount, it is necessary to clarify the spatial distribution of these released PTEs and understand the geochemical parameters that control it.

In the present study, a total of 50 samples were collected from a temporary storage pit where 100 m^3^ of rocks had been deposited immediately after excavation and investigated both the total PTE amounts and PTE amounts released from the rock material to clarify their spatial distribution. In addition, the geochemical parameters that control the spatial distribution of the released PTE content were determined based on the physicochemical characteristics of the excavated material. The obtained results will provide valuable information to design chemical immobilization treatments taking into consideration the spatial distribution of released PTE amounts.

## Materials and methods

### Preparation of the excavated shale samples

A total of 50 shale samples were collected from a temporary storage pit of rocks (100 m^3^) at a tunnel construction site on Kyushu Island, Japan, immediately after excavation at 100 m from the tunnel opening (Fig. S1, hereafter referred to as No. 1–50). They belonged to the Nichinan Group shale, which was formed by sedimentation and diagenesis during the Miocene period. The shale fragments were approximately 150–250 mm in diameter. The bulk-excavated samples were air-dried at room temperature for 1 week. A hammer with a rubber cover and a chisel were used to scrape 5–10 mm off the shale surface, removing the oxidized part from each sample. The shale samples were then crushed to particles with sizes < 0.5 mm using the chisel and hammer as well as an agate mortar for hand grinding. Adjustments were made to obtain a homogeneous particle size. The samples were stored at − 20 °C within 4 h from the start of surface removal to prevent oxidation. This study targeted arsenic, selenium, lead, and chromium as PTE because these PTEs include in the Japanese environmental quality standards for soil (Ministry of the Environment, 2011) although cadmium is also included in the standards, but was not detected from the rock samples.

### Analytical methods

All reagents with the analytical grade were purchased from FUJIFILM Wako Pure Chemical Co., Japan and KANTO CHEMICAL Co., Inc., Japan. Electrical conductivity (EC), oxidation–reduction potential (ORP), and pH values were measured in ultrapure water with a liquid/solid (L/S) ratio of 10/1 using a multiple water quality meter (MM-60R, DKK-TOA Co., Tokyo, Japan) with an EC cell (CT-47101B, DKK-TOA Co., Tokyo, Japan), ORP electrodes (PST-5721C, DKK-TOA Co., Tokyo, Japan), and pH electrodes (GST-5741C, DKK-TOA Co., Tokyo, Japan), respectively. The Eh values were converted from ORP values using the unit electrode correction value of the comparison electrode. The amounts of total arsenic, selenium, lead, and chromium in the shale samples were determined by the acid digestion method using a microwave digestion system (ETHOS EASY; Milestone, Italy) with 14.5 M HNO_3_ and 12 M HCl. The digested solution was passed through a 0.45-μm filter, and elemental concentration was measured using graphite furnace atomic absorption spectrometry (GFAAS; Z-5010, Hitachi, Ltd., Tokyo, Japan). The elements released from the shale sample were extracted using ultrapure water (L/S ratio of 10/1), passed through 0.45-μm filters, and then analyzed using 1) inductively coupled plasma mass spectrometry (ICP-MS; NexION 300P, PerkinElmer Co. Ltd., Waltham, MA, USA) for arsenic, selenium, lead, and chromium, 2) ion chromatography (IC) with an INTEGRION system and a Dionex IonPac™ CS16 and AS22 IC columns (Thermo Fisher Scientific Inc., USA) for cations and anions, respectively, 3) inductively coupled plasma optical emission spectrometry (ICP-OES; Optima 8300, PerkinElmer Co. Ltd., Waltham, MA, USA) for iron, aluminum, manganese, and silica, and 4) total organic carbon analysis (TOC-L_CPH_, Shimadzu Co., Japan) for water-soluble organic carbon (WSOC). External standards were used for calibration. A limit of detection for arsenic, selenium, lead, and chromium concentrations analysis by ICP-MS was 0.001, 0.001, 0.001, and 0.005 mg L^−1^, respectively. The relative 3σ standard deviations on repeat analysis were within 10%. The HCl-soluble arsenic, selenium, lead, and cadmium in the samples were extracted using 1 M HCl (L/S ratio of 100/3), passed through 0.45-μm filters, and then analyzed using GFAAS. Amorphous iron and aluminum were extracted using 0.2 M oxalate buffer at pH 3.0 (Shuman 1985), and were analyzed using ICP-OES. The loss on ignition (LOI) values were determined by thermally treating the samples at 750 °C. Mineralogical composition was analyzed using X-ray diffraction (XRD; MultiFlex, Rigaku Co., Japan) with Cu Kα radiation at 40 kV and 40 mA. The surfaces of shale particles were observed using a scanning electron microscope (SEM; JSM-7800 F, JEOL Co., Japan) to confirm the presence and distribution of pyrite in the samples.

### Collection of colloidal particles from water extracts

Colloidal particles were collected from the shale samples to evaluate their mineralogical composition and LOI. Samples No. 5, 7, 15, 20, 34, 36, 37, 38, 39, and 41, which contained high amounts of released iron, were mixed in equal quantities of 3.0 g each. The total 30-g sample was then shaken at 200 rpm for 24 h in ultrapure water at a L/S ratio of 10/1. After centrifugation at 5000 rpm for 5 min, the suspension was passed through a 0.45-μm filter. The filtrate was again centrifuged at 8000 rpm for 10 min, and the residue was collected and freeze-dried. The mineralogical composition and LOI value were determined using the previously mentioned procedures.

### Data analysis

The mean, maximum, and minimum values as well as the coefficients of variation (*CVs*) were obtained as descriptive statistics for the total and released PTE amounts from samples No. 1–50. Specifically, the *CV* values were obtained by dividing the standard deviation by the mean, as shown in Eq. 1. Based on the samples’ standard deviations, the relationship between sample size and data estimation error was analyzed using Eq. 2:

1 $$CV = s/M$$

2 $$D = ts/N^{0.5}$$

where *s* is the standard deviation, *M* is the sample mean, *D* is the estimation error, *t* is the *t*-value obtained from the *t* distribution table (*p* < 0.05), and *N* is the sample size. The ratio of this estimation error to the average value was defined as the error (%) according to the sample size, and the relationship between sample size and error was determined with a 95% confidence interval.

The geochemical factors controlling PTE release were investigated via multiple regression analysis using JMP software (ver. 8.0.2; SAS Institute, Inc., Cary, NC, USA). Standardization was performed by dividing the difference between average values and measured values by the standard deviation to align the units of the explanatory variables. The analysis was conducted using the stepwise method. The explanatory variables were selected based on elements that were soluble in water, 1 M HCl, and 0.2 M oxalate buffer. They were then determined by selecting factors with an *F* value ≥ 2.0 and a *P*-value ≤ 0.05 in descending order of *F* value.

The saturation index (SI) in the extracts of each excavated shale sample was calculated by inputting the elemental concentration as well as the pH and pe values of water extracts into the equilibrium geochemical modeling program PHREEQC Ver. 2.14.3 based on the minteq.v4 database (Parkhurst and Appelo, 2013). The pe values were calculated using the measured Eh values as shown in Eq. 3:

3 $$pe=\frac{F}{2.303RT}Eh$$

where *F* is the Faraday constant (96 485 coulombs mol^−1^), *R* is the gas constant (8.3143 J mol^−1^ K^−1^), and* T* is the absolute temperature (298 K).

## Results

### Total and released PTE amounts in the excavated shale samples

The total arsenic concentration in the excavated shale samples was 6.1 mg kg^−1^ on average, with maximum and minimum values of 17.0 and 3.3 mg kg^−1^, respectively (Table 1). The average, maximum, and minimum concentrations were 0.7, 2.2, and 0.1 mg kg^−1^ for selenium, 19.6, 28.6, and 12.0 mg kg^−1^ for lead, and 42.8, 65.0, and 23.1 mg kg^−1^ for chromium, respectively. High *CVs* were detected for the total amounts of arsenic, selenium, lead, and chromium, with values of 48.7%, 47.2%, 24.6%, and 22.7%, respectively.

**Table 1:** Descriptive statistics of the total contents of potentially toxic elements in the rock sample

Item	As	Se	Pb	Cr
Mean (mg kg^−1^)	6.1	0.7	19.6	42.8
Maximum (mg kg^−1^)	17.0	2.2	28.6	65.0
Minimum (mg kg^−1^)	3.3	0.1	12.0	23.1
Coefficient of variation (%)	48.7	47.2	24.6	22.7

The average content of water-soluble arsenic in the excavated samples was 0.37 mg kg^−1^, with maximum and minimum values of 0.70 and 0.01 mg kg^−1^, respectively (Table 2). The average, maximum, and minimum concentrations were 0.09, 0.18, and 0.02 mg kg^−1^ for selenium, 0.13, 0.24, and 0.01 mg kg^−1^ for lead, and 0.29, 0.52, and 0.01 mg kg^−1^ for chromium, respectively. High *CVs* were detected for the amounts of water-soluble selenium, arsenic, lead, and chromium, with values of 48.5%, 43.8%, 40.5%, and 39.4%, respectively.

**Table 2:** Descriptive statistics of the amounts of potentially toxic elements released from the rock samples

Item	As	Se	Pb	Cr
Mean (mg kg^−1^)	0.37	0.09	0.13	0.29
Maximum (mg kg^−1^)	0.70	0.18	0.24	0.52
Minimum (mg kg^−1^)	0.01	0.02	0.01	0.01
Coefficient of variation (%)	43.8	48.5	40.5	39.4

### Sample size and estimation error for the amount of released PTEs

**Fig. 1:**
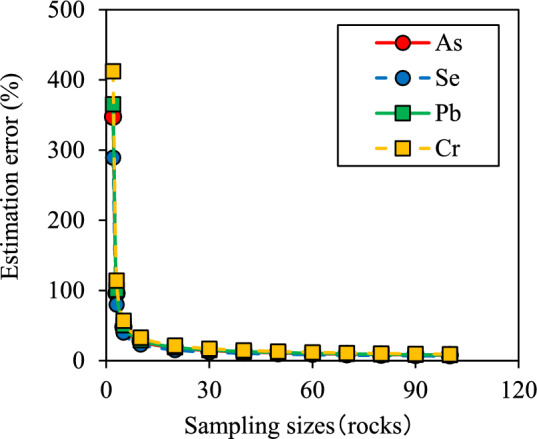
Relationship between the number of excavated shale samples and estimation error for the amount of potentially toxic elements released

Figure 1 shows the relationship between sample size and estimation error for the amounts of released water-soluble PTEs. Similar curvilinear relationships were observed even for different PTE types. The larger the sample size, the smaller the estimation error, and 250–400 shale samples were required to obtain an estimation error < 5%. The decrease in estimation error with the increasing sample size was especially reduced for sample sizes between 1 and 50 and between 10 and 20. The estimation errors for the amounts of water-soluble PTEs exhibited ranges of 97.0%–119.2%, 48.5%–59.5%, and 27.9%–34.3% for sizes of 3, 5, and 10 samples, respectively (Table S1). An increase in sample size from 3 to 10 reduced the estimation error by ca. 75%.

### Geochemical parameters to determine the amount of released PTEs

**Fig. 2:**
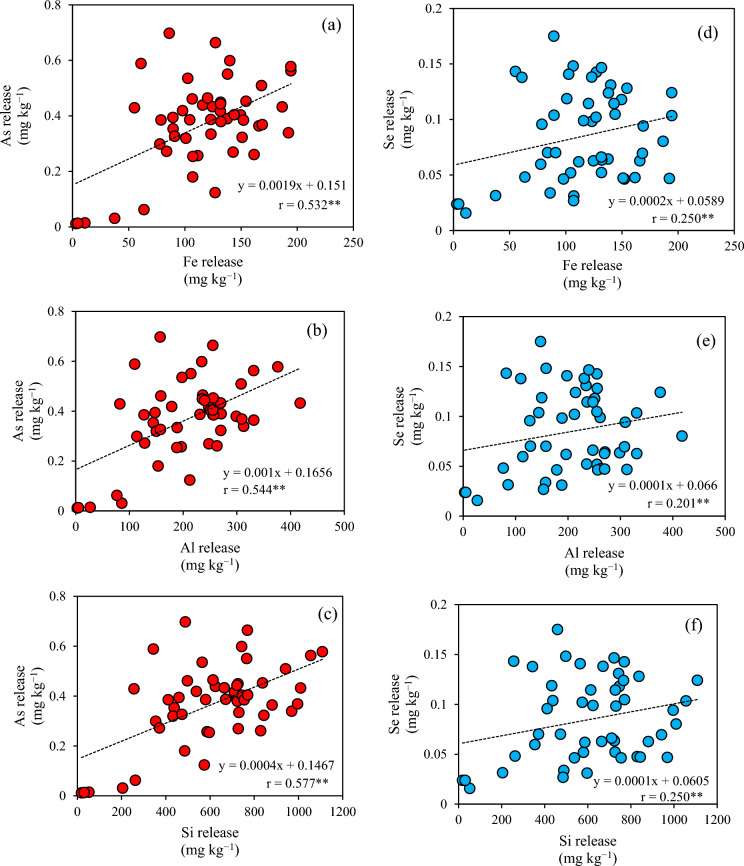

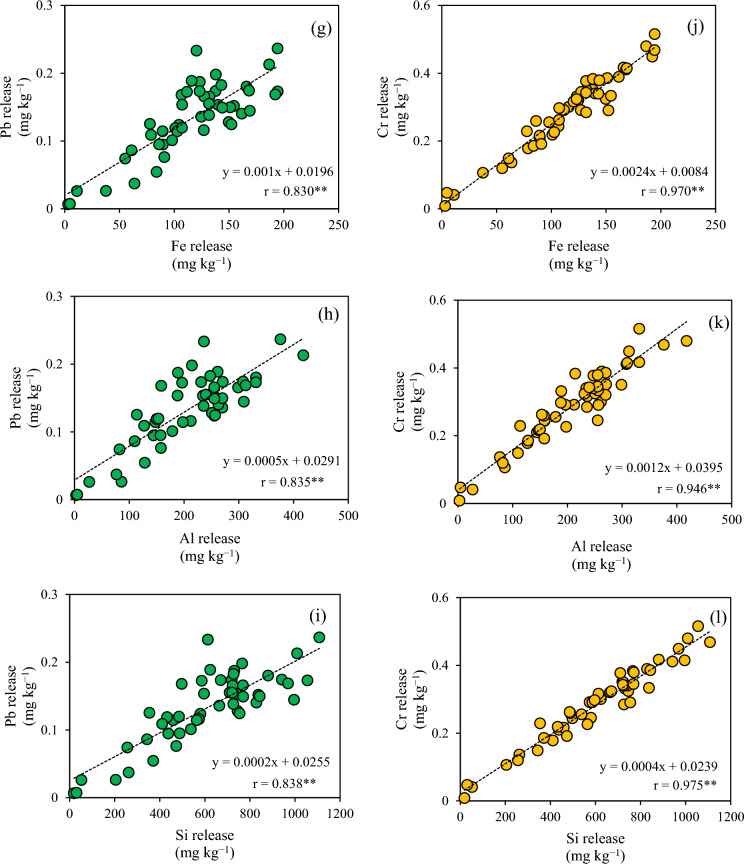
Relationship between iron, aluminum, and silicon and the amount of potentially toxic elements in water extracts: **a**–**c** arsenic, **d**–**f** selenium, **g**–**i** lead, and **j**–**l** chromium

**Fig. 3:**
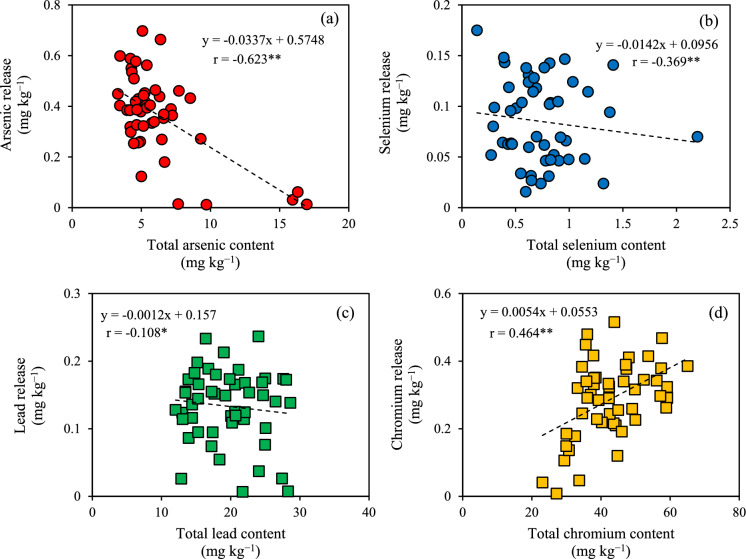
Relationship between the total and released amounts of potentially toxic elements: **a** arsenic, **b** selenium, **c** lead, and **d** chromium

The relationships between the amounts of released iron, aluminum, and silica and that of released PTEs were investigated via single regression analysis (Fig. 2). Positive linear correlations with high correlation coefficients were observed for lead and chromium, while no correlations were found for total PTE contents, regardless of PTE types (Fig. 3).

Due to the low correlation coefficients obtained via single regression analysis, multiple regression analysis was performed for the amounts of released arsenic and selenium. The amounts of amorphous iron, aluminum, released aluminum and manganese, and HCl-soluble arsenic were selected as significant explanatory variables, with a corrected multiple correlation coefficient of 0.778 (Table 3). The highest coefficient of − 6.7 × 10^−1^ was obtained for amorphous iron; thus, the higher the amount of amorphous iron in the rock samples, the lower the amount of arsenic released. The second highest coefficients were 3.8 × 10^−1^ and 2.9 × 10^−1^ for the amounts of released aluminum and HCl-soluble arsenic, respectively; thus, the higher their amounts in the rock samples, the higher the amount of arsenic released. The corrected multiple correlation coefficient for released selenium obtained via multiple regression analysis was 0.416, which was lower than that for released arsenic (Table 4). The highest coefficient of − 9.2 × 10^−1^ was obtained for amorphous iron; thus, the higher amount of amorphous iron in the rock samples, the lower the amount of arsenic released. The amounts of released sulfate and WSOC were selected as significant explanatory variables; thus, the higher their content in the rock samples, the higher the amount of selenium released.

**Table 3:** Multiple regression analysis testing the relationship between water-soluble arsenic and geochemical parameters in the excavated shale samples

	Coefficient	*t* value	*p* value
Intercept	− 4.81 × 10^−11^	0.00	1.000
Amorphous iron	− 6.7 × 10^−1^	− 7.18	< 0.0001
Aluminum release	3.8 × 10^−1^	3.32	0.0018
HCl-soluble arsenic	2.9 × 10^−1^	3.63	0.0007
Amorphous aluminum	− 2.7 × 10^−1^	− 3.85	0.0004
Manganese release	− 2.6 × 10^−1^	− 2.72	0.0094
Coefficient of determination	0.800		
Adjusted coefficient of determination	0.778		
*F* value	35.3		
AIC	88.8

**Table 4:** Multiple regression analysis testing the relationship between water-soluble selenium and geochemical parameters in the excavated shale samples

	Coefficient	*t* value	*p* value
Intercept	3.3 × 10^−11^	0.00	1.000
Amorphous iron	− 9.2 × 10^−1^	− 5.29	< 0.0001
Sulfate release	6.2 × 10^−1^	3.36	0.0016
WSOC	3.8 × 10^−1^	3.45	0.002
Magnesium release	2.4 × 10^−1^	− 2.02	0.049
Coefficient of determination	0.464		
Adjusted coefficient of determination	0.416		
*F* value	9.7		
AIC	123.7		
WSOC: water-soluble organic carbon			

### Mineralogical compositions in the shale and colloidal particles collected from water extracts

**Fig. 4:**
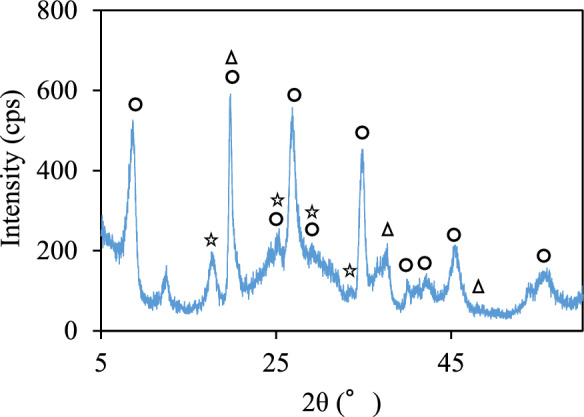
XRD profile for the colloidal particles. The circle, star, and triangle indicate: 〇: (K_0.7_Ca_0.13_)(Mg_0.6_Al_1.3_CrO_2_Fe_0.1_)(Al_0.9_Si_3.1_O_10_)(OH)_2_. ☆: NaFe(CrO_4_)_2_(H_2_O)_2_, NaAl(CrO_4_)_2_(H_2_O)_2_. △: CrO(OH)

The mineralogical compositions identified via XRD analysis were almost the same among the rock samples examined in this study, but the first peaks for siderite and pyrite, i.e., 2.80 and 2.72 Å, respectively, had different strengths depending on the samples (Fig. S2). SEM observations confirmed that the observed pyrite was framboidal, with different amounts being detected in different samples (Fig. S3). Chromium-containing crystalline minerals were identified in the colloidal particles with size over 0.45-μm collected from the water extracts (Fig. 4).

The predicted SI of ferrihydrite, goethite, hematite, gibbsite, boehmite, halloysite, and hercynite in the water extracts was supersaturated, while that of ferric arsenate was unsaturated (Table 5). The predicted SI of iron chromite was slightly supersaturated in the water extracts from No. 1, 10, 17, 19, 30, and 50 samples.

**Table 5:** Saturation index values for each excavated shale sample via the equilibrium geochemical modeling program PHREEQC

Parameter	No.1	No.9	No.10	No.17	No.19	No.21	No.27	No.30	No.49	No.50
pH (measured)	8.64	10.08	8.54	8.65	9.31	9.67	8.51	9.03	9.56	8.35
pe (calculation from Eh)	5.49	4.44	5.39	5.33	4.90	4.71	5.49	4.80	5.00	5.71
FeAsO_4_: 2H_2_O	− 8.66	− 9.95	− 7.61	− 7.29	− 8.30	− 8.88	− 9.05	− 7.41	− 8.88	− 8.50
Ferrihydrite (Fe(OH_3_)	4.45	4.60	4.99	5.21	5.24	5.03	3.89	5.21	4.96	4.07
Goethite (FeOOH)	7.15	7.30	7.69	7.91	7.94	7.73	6.89	7.91	7.65	6.77
Hematite (Fe_2_O_3_)	16.70	17.00	17.77	18.21	18.27	17.85	15.58	18.22	17.77	15.95
Gibbsite (Al(OH)_3_)	1.73	1.29	2.33	2.18	1.95	1.67	0.89	2.02	1.56	1.28
Boehmite (AlOOH)	1.44	1.00	2.04	1.89	1.67	1.38	0.81	1.73	1.26	1.00
FeCr_2_O_4_	0.09	− 6.25	2.5	2.64	− 0.44	− 3.17	− 0.98	2.51	− 4.23	0.72
Halloysite (Al_2_Si_2_O_5_(OH)_4_)	2.94	3.41	5.34	5.25	5.30	4.70	0.39	5.15	4.22	1.62
Hercynite (FeAl2O4)	3.69	2.57	5.62	5.5	4.85	3.89	1.58	5.32	3.46	2.50
Massicot (PbO)	− 4.67	− 3.13	− 4.77	− 4.52	− 3.48	− 3.10	− 5.39	− 4.01	− 3.33	− 5.53
PbCrO_4_	− 3.78	− 4.13	− 3.62	− 3.28	− 2.98	− 3.24	− 5.20	− 3.29	− 3.36	− 4.40
Pb(OH)_2_	0.07	1.61	− 0.03	0.23	1.26	1.64	− 0.65	0.74	1.41	− 0.78

## Discussion

### Spatial distribution of total PTE amounts in the excavated rocks at the temporary storage pit and PTE contents released from them

The *CVs* for the total PTE amounts in the excavated rocks depended on PTE types. For example, values were higher for arsenic and selenium than for lead and chromium (Table 1). Arsenic and selenium are contained in pyrite as impurities (Matamoros-Veloza et al., 2014; Kamata & Katoh, 2019; Ueshima et al., 2019). The amounts of framboidal pyrite detected via SEM observations varied depending on the rock samples (Fig. S3). The heterogeneous distribution of these PTE-containing minerals among the rock samples would attribute to the higher *CVs* for total arsenic and selenium. A previous study showed that the *CVs* for total PTE amounts obtained from 352 samples within an area of 4,789 km^2^ were 34.0%, 74.0%, and 23.0% for arsenic, lead, and chromium, respectively (Yang et al., 2011). Similarly, those obtained for total arsenic and lead from 413 samples within an area of 1,620 km^2^ were 133.0% and 43.0%, respectively (Wang et al., 2019). In previous studies, the spatial distribution of total PTE amounts was obtained based on large-scale sampling ranges. The present study indicated that the *CVs* for the total PTE amounts obtained from 100 m^3^ of excavated rock at the temporary storage pit were comparable with those obtained from an area of several thousand km^2^. The spatial distribution of total PTE amounts may be similar at sites with the same geological formation.

The *CV* values for the total and released PTE amounts did not differ significantly (Tables 1 and 2). However, no clear correlation was detected between the two amounts for any PTE type (Fig. 3). This suggested that the amount of PTE released from the excavated rock depended on the elements’ phases and geochemical characteristics, and not on the total PTE amount. At the tunnel construction site, the amounts of released PTEs are periodically evaluated to determine whether the excavated rocks pose potential environmental risks. Environmental risks are assessed once for every specific volume of rocks placed in the temporary storage pit (e.g., 100–5,000 m^3^). This is because rocks are excavated in large quantities, and frequent evaluation is cost- and time-effective. Accordingly, it is important to estimate the range of PTE amounts released from a small number of rock samples stored in the pit based on the estimation error in order to accurately design chemical immobilization treatments. Based on the results obtained in this study, the number of samples that need to be analyzed to obtain estimation errors < 5% and 10% are 250–400 and 100, respectively (Fig. 1). Hundreds of rock samples are difficult to analyze, although the smaller the estimation error, the more narrowly the amount of released PTEs can be estimated. In this study, the *CV* for such amount ranged from 39.4 to 48.5% (Table 2). For any PTE type, the number of rock samples required to obtain an estimation error within 30% was shown to be 10 (Fig. 1), assuming that the amount of PTEs released from the rocks stored in the temporary storage pit exhibits this level of variability. This implies that the environmental risk can be performed using the preparation of 10 mixed rock samples collected from the pit, leading to estimations of released PTE amounts with an error of 30%. Thus, the estimation of the amount of PTE released 30% more allows us to conservatively estimate the total amount of PTEs released within the excavated rocks stored in the pit. Then, the appropriate amount of immobilization material that should be used to completely prevent arsenic leaching can be calculated based on the total PTE amount released within the excavated rocks.

### Geochemical parameters to determine the spatial distribution of released PTEs

Strong positive linear relationships were found between the amounts of released lead and chromium and those of released iron, aluminum, and silica (Fig. 2). The predicted SI value of minerals containing iron, aluminum, and silica in the water extracts was supersaturated (Table 5). This suggested that the contents of lead and chromium released from the excavated shale were controlled by the amounts of colloidal particles under 0.45 μm of inorganic minerals such as ferrihydrite, goethite, hematite, gibbsite, boehmite, halloysite, and hercynite. The predicted SI value of iron chromite was slightly supersaturated in the water extracts of No. 1, 10, 17, 19, 30, and 50 samples, which contained high amounts of amorphous iron. Chromium (VI) is reduced to chromium (III) by the oxidation of iron (II) dissolved from iron carbonate, resulting in the precipitation of iron chromite (Bibi et al., 2018; Dong et al., 2011; Erdem et al., 2004; Tang & Martin, 2011). In contrast to chromium, the predicted SI value of minerals containing lead, iron, aluminum, and silica was unsaturated in the water extracts, but that of lead hydroxide was supersaturated in most samples. Lead would be released with the sorption on the surface of inorganic colloidal particles with size under 0.45-μm. Both lead and chromium are transported by these particles through solid pores (Pokrovsky & Schott, 2002). The results of this study provide new insights into the release of lead and chromium from the excavated rocks via inorganic colloidal particles with size under 0.45-μm.

The amounts of released arsenic depended on the amounts of amorphous iron present in the rocks (Table 3). Rock samples with higher amounts of amorphous iron would account for lower amounts of released arsenic. The pH of excavated rocks affects PTE release, as its variation modifies the charge balance affecting the PTE sorption ability (Hingston et al., 1972; Ma et al., 2015; Raven et al., 1998). However, pH was not selected as an explanatory variable in the multiple regression analysis in this study because of the multicollinearity produced by the strong correlation between pH and amorphous iron (Table S2). The rock samples with high amounts of amorphous iron contained siderite and pyrite (Fig. S2). The dissolution of siderite and the oxidation of pyrite result in the precipitation of iron hydroxide and release of hydrogen ions. In addition, siderite accelerates the rate of pyrite oxidation (Caldeira et al., 2010). Iron hydroxide, which is likely extracted as amorphous iron, has a large specific surface area and provides the sorption sites for arsenic (Pierce & Moore, 1982). Thus, the two above-mentioned processes would lead to the precipitation of iron hydroxide, resulting in the decrease in pH and increase in the amount of amorphous iron. Ultimately, the precipitation of iron hydroxide would enhance the sorption ability of arsenic in the excavated rocks. After amorphous iron, the second largest contributor to the release of arsenic was the amount of released aluminum. The predicted SI of gibbsite and boehmite was supersaturated in the water extracts (Table 5), suggesting that the higher the amounts of released aluminum, the higher the arsenic release in conjunction with the complexation with inorganic colloidal particles such as gibbsite and boehmite. The third largest contributor to arsenic release was the amount of HCl-soluble arsenic. The arsenic sorbed on the surface of solid minerals such as iron hydroxide and pyrite would be extracted as HCl-soluble arsenic, suggesting that as HCl-soluble arsenic increases, so does the amount of released arsenic.

The amounts of released selenium also depended on the amorphous iron content (Table 3). As observed for arsenic, the sorption sites for selenium provided by higher amorphous iron content would account for lower amounts of selenium being released. After amorphous iron, the second largest contributor to selenium release was the amount of released sulfate. This was selected as an explanatory variable because of the sorption competition between selenium and sulfate (Ramana & Sengupta, 1992). The third largest contributor to selenium release was the amount of released WSOC. Bassil et al. (2018) reported that the presence of humic-like organic matter increased the release of selenium under alkaline pH conditions due to the co-dissolution or decomplexation between it and water-soluble organic matter (WSOM). Thus, the release of selenium from rock samples containing high amounts of WSOC would increase due to the complexation with WSOM and selenium as well as to the sorption competition between them.

As observed for the *CVs* obtained for the amounts of released PTEs, the geochemical parameters controlling these contents were similar between arsenic and selenium as well as between lead and chromium. In addition, the above-mentioned *CVs* were higher for the former two elements than for the latter (Table 2). The amount of amorphous iron exerted the strongest control on the release of arsenic and selenium from the excavated rocks, with complex mechanisms regulating both their release and retention. For example, the process of iron hydroxide dissolution/precipitation has been shown to lead to arsenic and selenium release/retention, respectively (Tabelin et al., 2017). Furthermore, increases in pH accelerate the release of these two elements (Tabelin et al., 2014). Multiple regression analyses have shown that other geochemical factors also influence the release of arsenic and selenium. In contrast, the mechanisms underlying the release of lead and chromium from excavated rocks are simpler. Simple regression analyses have shown that the release of inorganic colloidal particles containing iron, aluminum, and silica increases that of lead and chromium. Therefore, the difference in *CVs* depending on PTE types would be attributed to whether the geochemical parameters controlling PTE release are mainly present in the solid or liquid phase. The present study suggested that the lower the amounts of framboidal pyrite and inorganic colloidal particles with size under 0.45-μm, the lower the heterogeneity of the PTE released from the excavated rocks. The amounts of PTE release from such excavated rock would differ only slightly from the estimated amounts of released PTEs. The oxidation of framboidal pyrite in the excavated rocks will increase the amount of amorphous iron and decrease the rocks’ pH, thus altering the *CVs* for the released PTE amounts. Future studies should investigate the heterogeneous distribution of released PTEs after atmospheric exposure because environmental risk assessments are normally carried out using excavated rocks that are stored at temporary storage pits for several weeks after excavation.

## Conclusions

The *CVs* for the amounts of total arsenic, selenium, lead, and chromium in a volume of 100 m^3^ of excavated shale stored at a temporary storage pit were 48.7%, 47.2%, 24.6%, and 22.7%, respectively. Similarly, those of released arsenic, selenium, lead, and chromium were 43.8%, 48.5%, 40.5%, and 39.4%, respectively. The *CVs* for the total PTE amounts and released PTE amounts were similar and depended on the PTE type; however, no linear relationship between them was detected. A sample of 10 rocks had an estimation error of ± 30%. The geochemical parameters controlling the amounts of PTEs released from the excavated shale differed depending on the PTE type. Multiple regression analysis showed that the release of arsenic and selenium was mainly controlled by the amount of amorphous iron. The higher the content of amorphous iron, the lower the amounts of released arsenic and selenium. Simple regression analysis indicated that the release of lead and chromium was controlled by the amounts of inorganic colloidal particles with size under 0.45-μm. The higher the content of these particles, the higher the amounts of lead and chromium released. This study suggested that the preparation of 10 mixed rock samples collected from temporary storage pits leads to an estimation of released PTE amounts with a 30% error. In addition, the actual PTE amount released from excavated rocks containing high and low amounts of amorphous iron and inorganic colloidal particles with size under 0.45-μm, respectively, would differ little from the estimated amounts. Future studies should investigate how the heterogeneous distribution of released PHEs is altered after atmospheric exposure (e.g., rainfall, temperature, and oxidation).

## Supplementary Information

Below is the link to the electronic supplementary material.

Supplementary file1 (DOCX 185 KB)

## Data Availability

All used data are presented in tables and figures in the paper. No datasets were generated or analysed during the current study.
