# Supplementary material for: Determination of geochemical parameters that control the spatial distribution of potentially toxic elements released from excavated shale at a temporary storage pit
Source: Environ Geochem Health. 2025 Jun 10;47(7):259. doi: 10.1007/s10653-025-02577-8 (PMC12152043; doi:10.1007/s10653-025-02577-8)
Supplement: Supplementary file 1 — Supplementary file1 (DOCX 185 KB) [file 10653_2025_2577_MOESM1_ESM.docx]

-Supplementary Data-

Article in Environmental Geochemistry and Health

Determination of geochemical parameters that control the spatial distribution of potentially toxic elements released from excavated shale at a temporary storage pit

Shoji Suzuki^a, 1^, Toshihiko Miura^b^, Kenichi Ito^c^, Masahiko Katoh^d, *^

^a^Department of Agricultural Chemistry, Graduate School of Agriculture, Meiji University, 1-1-1, Higashimita, Tama, Kawasaki, Kanagawa 214-8571, Japan

^b^Technical Research Institute, Obayashi Corporation, 4-640 Shimokiyoto, Kiyose-shi, Tokyo 204-8558, Japan

^c^Center for Collaborative Research and Community Cooperation, the University of Miyazaki, 1-1, Gakuen Kibanadai-nishi, Miyazaki, Miyazaki 889-2192, Japan

^d^Department of Agricultural Chemistry, School of Agriculture, Meiji University, 1-1-1, Higashimita, Tama, Kawasaki, Kanagawa 214-8571, Japan

^1^ Present address: Technical Research Institute, Okumura Corporation, 387 Ohsuna, Tsukuba, Ibaraki 300-2612, Japan

* Corresponding author: Masahiko Katoh

Department of Agricultural Chemistry, School of Agriculture, Meiji University, 1-1-1, Higashimita, Tama, Kawasaki, Kanagawa 214-8571, Japan

*Tel:* +81 44 934 7101; *Fax:* +81 44 934 7902; *Email address:* [mkatoh@meiji.ac.jp](mailto:mkatoh@meiji.ac.jp)

ORCID: 0000-0001-6219-2050

| **Table S1**　Estimation error of pH, EC, and amount of potentially toxic elements released in analysis of 3, 5, and 10 rocks | | | | | | |
| --- | --- | --- | --- | --- | --- | --- |
|  | pH | EC | As | Se | Pb | Cr |
| Error at 10 rock masses (%) | 3.3 | 9.4 | 31.0 | 34.3 | 28.7 | 27.9 |
| Error at 5 rock masses (%) | 5.6 | 16.2 | 53.8 | 59.5 | 49.8 | 48.5 |
| Error at 3 rock masses (%) | 11.3 | 32.5 | 107.6 | 119.2 | 99.6 | 97.0 |
| EC: electrical conductivity | | | | | | |

| **Table S2** Correlation coefficients of physicochemical properties of excavated shale in No.1-50 | | | | | | | | | | | | | | | | |
| --- | --- | --- | --- | --- | --- | --- | --- | --- | --- | --- | --- | --- | --- | --- | --- | --- |
|  | pH | EC | Cl^-^ | SO_4_^2-^ | Na^+^ | K^+^ | Mg^2+^ | Ca^2+^ | Fe | Al | Mn | Si | Am-Fe | Am-Al | LOI | WSOC |
| pH |  |  |  |  |  |  |  |  |  |  |  |  |  |  |  |  |
| EC | −0.322* |  |  |  |  |  |  |  |  |  |  |  |  |  |  |  |
| Cl^-^ | 0.017 | 0.023 |  |  |  |  |  |  |  |  |  |  |  |  |  |  |
| SO_4_^2-^ | −0.722** | 0.164 | 0.262 |  |  |  |  |  |  |  |  |  |  |  |  |  |
| Na^+^ | −0.313* | 0.837** | 0.106 | 0.164 |  |  |  |  |  |  |  |  |  |  |  |  |
| K^+^ | −0.640** | 0.597** | 0.129 | 0.570 | 0.526** |  |  |  |  |  |  |  |  |  |  |  |
| Mg^2+^ | −0.039 | −0.277* | 0.015 | 0.361 | −0.136 | 0.127 |  |  |  |  |  |  |  |  |  |  |
| Ca^2+^ | −0.316* | 0.368** | 0.221 | 0.191 | 0.060 | 0.491** | −0.268 |  |  |  |  |  |  |  |  |  |
| Fe | 0.698** | −0.531** | 0.010 | −0.549** | −0.442** | −0.497** | −0.125 | −0.126 |  |  |  |  |  |  |  |  |
| Al | 0.682** | −0.526** | −0.036 | −0.512** | −0.528** | −0.488** | −0.111 | −0.037 | 0.952** |  |  |  |  |  |  |  |
| Mn | 0.431** | −0.090 | 0.060 | −0.498** | 0.017 | −0.206 | −0.253 | 0.022 | 0.793** | 0.689** |  |  |  |  |  |  |
| Si | 0.710** | −0.568** | 0.014 | −0.503** | −0.493** | −0.507** | −0.093 | −0.129 | 0.985** | 0.961** | 0.710** |  |  |  |  |  |
| Am-Fe | −0.966** | 0.395* | −0.005 | 0.747** | 0.354* | 0.762** | 0.072 | 0.372** | −0.702** | −0.670** | −0.452** | −0.703** |  |  |  |  |
| Am-Al | −0.203 | −0.240 | −0.100 | 0.089 | −0.228 | 0.141 | 0.365** | −0.080 | −0.081 | −0.095 | −0.077 | −0.112 | 0.216 |  |  |  |
| LOI | −0.941** | 0.421** | 0.022 | 0.658** | 0.426** | 0.724** | 0.092 | 0.280 | −0.705** | −0.706** | −0.412** | −0.721** | 0.958** | 0.242 |  |  |
| WSOC | −0.063 | 0.296* | −0.114 | −0.039 | 0.084 | −0.026 | −0.098 | 0.075 | −0.339* | −0.291* | −0.275 | −0.358* | 0.067 | 0.093 | 0.029 |  |
| Significance level correlation coefficient, **: p<0.01, *: p<0.05 | | | | | | | | | | | | | | | | |


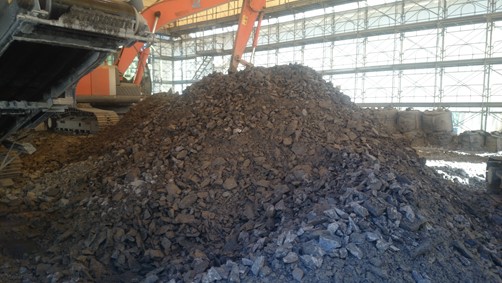


**Fig. S1** Temporally storage pile at a tunnel construction site


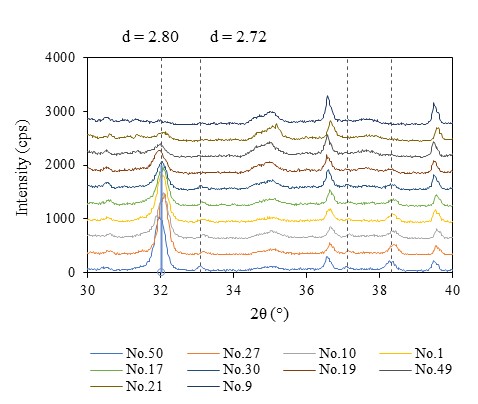


**Fig. S2** XRD profiles for No.1, 9, 10, 17, 19, 21, 27, 30, 49, and 50 samples.


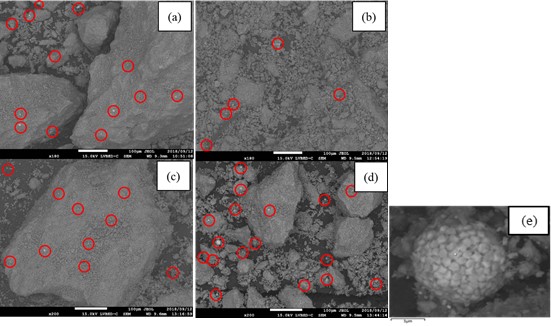


**Fig. S3** Presence of pyrite by SEM observation in No.1, 9, 49, and 50 samples. Red circles in the photograph indicate presence of pyrite. (a): No.1 sample, pH 8.6, (b): No.9 sample, pH 10.1, (c): No.49 sample, pH 9.6, (d): No.50 sample, pH 8.4, (e): framboidal pyrite.
